# Botulinum Neurotoxin Application to the Severed Femoral Nerve Modulates Spinal Synaptic Responses to Axotomy and Enhances Motor Recovery in Rats

**DOI:** 10.1155/2018/7975013

**Published:** 2018-09-05

**Authors:** Marcel Irintchev, Orlando Guntinas-Lichius, Andrey Irintchev

**Affiliations:** Department of Otorhinolaryngology, Jena University Hospital, Am Klinikum 1, 07747 Jena, Germany

## Abstract

Botulinum neurotoxin A (BoNT) and brain-derived neurotrophic factor (BDNF) are known for their ability to influence synaptic inputs to neurons. Here, we tested if these drugs can modulate the deafferentation of motoneurons following nerve section/suture and, as a consequence, modify the outcome of peripheral nerve regeneration. We applied drug solutions to the proximal stump of the freshly cut femoral nerve of adult rats to achieve drug uptake and transport to the neuronal perikarya. The most marked effect of this application was a significant reduction of the axotomy-induced loss of perisomatic cholinergic terminals by BoNT at one week and two months post injury. The attenuation of the synaptic deficit was associated with enhanced motor recovery of the rats 2–20 weeks after injury. Although BDNF also reduced cholinergic terminal loss at 1 week, it had no effect on this parameter at two months and no effect on functional recovery. These findings strengthen the idea that persistent partial deafferentation of axotomized motoneurons may have a significant negative impact on functional outcome after nerve injury. Intraneural application of drugs may be a promising way to modify deafferentation and, thus, elucidate relationships between synaptic plasticity and restoration of function.

## 1. Introduction

Injury to peripheral nerves in adult mammals causes deafferentation of the axotomized motoneurons, a phenomenon known as “synaptic stripping” [1]. Synaptic terminals are removed from cell bodies and dendrites of motoneurons by activated microglial and astroglial cells [1–6]. The overall posttraumatic loss is reversed to a large extent if muscles become reinnervated [3, 6, 7], but restoration of some synaptic inputs is incomplete [8–11]. Such deficits, for example, in cholinergic and glutamatergic innervation, may contribute to functional deficits after muscle reinnervation as they are well correlated with functional performance after long-term reinnervation [9, 12].

Here, we pursued to influence synaptic responses after peripheral nerve injury and, thus, eventually alter the outcome by using botulinum neurotoxin A (BoNT) or brain-derived neurotrophic factor (BDNF). When applied intramuscularly, BoNT blocks synaptic transmission at the neuromuscular junction and, in addition, is transported retrogradely to the motoneuron cell body and possibly also transcytosed to afferent synaptic terminals [13–16]. BoNT causes progressive synaptic stripping detectable at 4 days after intramuscular injection and abolishes excitatory and inhibitory synaptic transmission on motoneurons at 1-2 weeks after application [17]. Rather than intramuscularly, we applied BoNT to the proximal nerve stump immediately after nerve transection similar to the application of retrograde tracers assuming that this type of application will enhance synaptic stripping similar to intramuscular BoNT application. In other animals, we applied BDNF to the proximal stump of the freshly cut nerve hoping to achieve an effect opposite to that of BoNT, that is, attenuation of synaptic loss. When administered to cut proximal axons immediately after transection, BNDF reduces synaptic stripping and enhances recovery of tonic firing of regenerating motoneurons [18]. Synaptotrophic effects of exogenous BDNF have also been reported after ventral root avulsion [19]. Finally, a single session of brief electrical stimulation (20 Hz, 1 hour) of the proximal stump of the freshly transected femoral nerve in rats leads to enhanced nerve regeneration over weeks and this effect is apparently associated with an upregulation of BDNF and its cognitive receptor TrkB in the motoneuron cell body [20, 21]. It is possible, though not proven, that this enhanced BDNF signaling leads to, among other mechanisms, better regeneration via synaptotrophic effects. We measured the effects of BoNT or BDNF application using stereological estimates of chemically defined nerve terminal densities in motor nuclei, a motor recovery test, and retrograde labeling of motoneurons. For this first experiment using intraneural drug application, we selected the femoral nerve model in rats for a practical reason: the anatomy in this model allows work with a longer proximal trunk after nerve transection as compared with, for example, the facial nerve and, thus, easier application of BoNT or BDNF solutions to the severed nerve using plastic mini cups. The well-established femoral nerve model is a valuable alternative to other spinal nerve models like the sciatic one offering the possibility to analyze precision of target reinnervation, reliable functional assessments, and a straightforward search of anatomical deficits and structure-function correlations [22]. Helpful for this study was also previous data on long-term functional recovery, precision of motor reinnervation, and correlations between these measures after section/suture of the femoral nerve in adult rats [23].

## 2. Materials and Methods

### 2.1. Animals and Experimental Design

Ten-week-old female Wistar Unilever rats (*N* = 65) from Charles River Laboratories (Sulzfeld, Germany) were used. To monitor short-term numerical changes in synaptic terminal populations, retrograde neuronal tracer (Fluoro-Gold, FG) was injected unilaterally into the quadriceps muscles of 20 animals (experiment I). Four days later, the femoral nerve on the injected side was cut and solutions containing bovine serum albumin (BSA), BoNT, or BDNF were applied to the proximal nerve stump (5 rats per group, see details on application below). Synaptic populations in the quadriceps motor nucleus, defined by the retrograde labeling, were studied one week after nerve transection. The rest five rats served as an “intact” control, that is, they were similarly treated and analyzed with the exception of nerve injury. To analyze long-term synaptic alterations, the rats in experiment II were subjected to nerve lesion and application of BSA (*N* = 6), BoNT type A (*N* = 7), or BDNF (*N* = 7). Intramuscular (i.m.) injections of FG were performed two months after injury followed by, one week later, video recordings for single-frame motion analysis (SFMA) and tissue sampling for synaptic terminal analyses. Analysis of long-term functional effects was done in experiment III. After nerve injury and application of BSA (*N* = 7), BoNT (*N* = 10), or BDNF (*N* = 8), the animals were repeatedly video recorded over a 20-week observation period and then subjected to retrograde labeling of motoneurons regenerated beyond the injury site to analyze “preferential motor reinnervation” [24]. The animals were housed under standard conditions and received food and water ad libitum. Visual examinations for complications like BoNT-induced muscle paralysis, abnormal grooming, or self-mutilations were performed regularly (once daily in the first week, once or twice weekly at later time periods). Such complications were not observed. Experiments were performed according to the animal protection laws of Germany and the European Community. Experiments were blinded.

### 2.2. Surgery and Drug Application

**Figure 1:**
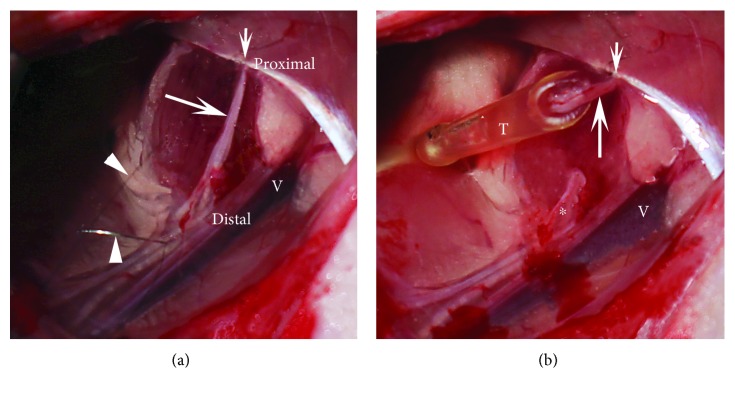
Drug application to the severed nerve. (a) The right femoral nerve trunk (arrow) prior to nerve injury. Proximally, the nerve is fixed by an epineural suture (short arrow) to the nearby muscle aponeurosis to prevent withdrawal of the proximal stump after nerve cut. Seen are also the 10–0 thread (upper arrowhead) used to fix the nerve and its needle (lower arrowhead), as well as the femoral vein (V). (b) The femoral nerve is transected, and the proximal stump is inserted in a self-made cup (T, see Materials and Methods) filled with drug solution. The distal nerve stump is marked by an asterisk.

Rats were anesthetized with fentanyl (Fentanyl Janssen, Janssen, Neuss, Germany, 0.005 mg/kg i.m.), midazolam (Dormicum-R, Roche, Basel, Switzerland, 2 mg/kg i.m.), and medetomidine (Domitor-R, Orion Pharma, Espoo, Finland, 0.15 mg/kg i.m.). The trunk of the right nerve was exposed under an operation microscope and cut at approximately 7 mm proximal to the bifurcation of the saphenous and quadriceps muscle branches (Figure 1(a)). The proximal nerve stump was inserted for 30 min into a cup containing 0.1% BSA (Sigma, Taufkirchen, Germany) in saline, 100 U/ml BoNT (Xeomin, Merz Pharma, Frankfurt, Germany), or 20 *μ*g/ml human recombinant BDNF (Biomol, Hamburg, Germany) in 0.1% BSA saline (Figure 1(b)). As a rough orientation for the drug concentrations served previous in vivo studies on synaptic effects using BoNT [13, 17] and BDNF [18]. The cups were cut from standard yellow pipette tips after their distal ends were heat-sealed using a lighter (Figure 1(b), capacity ~10 *μ*l). After drug treatment, the nerve trunks and their surroundings were thoroughly rinsed with saline and the nerve ends were aligned using two epineural 10–0 sutures (Ethicon, Norderstedt, Germany). Finally, the skin was closed with 4–0 sutures (Ethicon) and the rats received subcutaneously an antidote cocktail consisting of atipamezole (Antisedan, Orion Pharma, 0.75 mg/kg), flumazenil (Anexate, Roche, 0.2 mg/kg), and naloxone (Naloxon, CuraMed Pharma, Karlsruhe, Germany, 0.12 mg/kg).

### 2.3. Single-Frame Motion Analysis (SFMA)

SFMA was performed as described previously [23]. Briefly, the rats (experiments II and III) were video recorded prior to nerve injury from behind and from the left and right side during walking along a wooden plate (1500 mm long, 120 mm wide, and 20 mm thick) using a video camera (100 frames per second, Pike F-032, Allied Vision Technologies, Stadtroda, Germany). The video recordings were repeated 8 weeks (experiment II) or at 1, 2, 4, 8, 12, 16, and 20 weeks (experiment III) after injury. At least three walking trials were recorded per rear, left and right side view of each animal per time point. Analyses were performed using noncommercial software packages: VirtualDub 1.6.19 (http://www.virtualdub.org) and Image Tool 3.0 (University of Texas Health Science Center at San Antonio, TX, USA, http://compdent.uthscsa.edu/imagetool.asp). Two parameters were measured: the foot-base angle (FBA) and the step length ratio (SLR). The FBA is measured at toe-off position on the side ipsilateral to injury as an angle between the line dividing the sole surface into two halves and the horizontal line (minimum of 3 measurements per animal and time point). The SLR is calculated as ratio of the lengths of two successive steps (minimum of 6 SLR values per animal and time point). Using the FBA and SLR values, two additional parameters were calculated: (1) the product FBA × SLR and (2) the FBA × SLR recovery index [23].

### 2.4. Retrograde Labeling of Motoneurons

**Figure 2:**
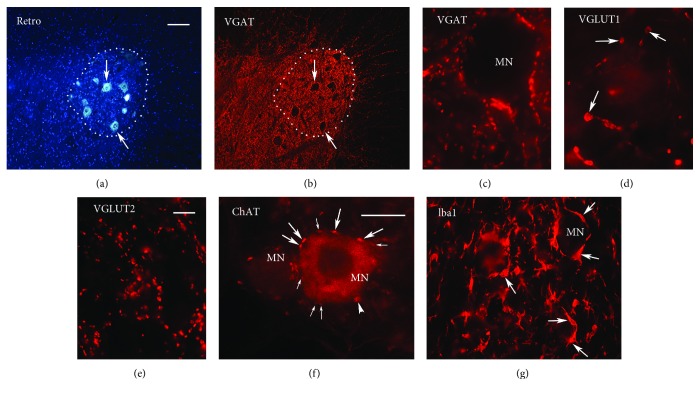
Images of synaptic terminals and Iba1^+^ cells in the quadriceps motor nucleus. (a-b) A section containing back-labeled cell bodies of femoral motoneurons (a, arrows) is additionally stained for nuclei (a) and VGAT (b). The boundary of the quadriceps motor nucleus is indicated by a dotted line. Scale bar = 100 *μ*m for (a-b). (c–e) VGAT^+^ and VGLUT 2^+^ axonal terminals (c, e) and VGLUT1^+^ varicosities (arrows, d). Scale bar = 10 *μ*m for (c–e). (f) ChAT staining of two motoneuron cell bodies (MN) surrounded by cholinergic terminals (arrows). Counted were terminals around the MN soma with a visible nucleus (pale area in the center of the MN on the right hand side) which were in focus (thick arrows). Terminals out of focus or only partially seen in the focus plane (thin arrows) were not counted. No quantification was undertaken for the second MN profile (on the left hand side) since it had no visible nucleus. The arrowhead points to a ChAT^+^ cross-sectional profile of a dendrite close to the MN cell body. Such “perisomatic” dendritic profiles could be traced for long distances throughout the section thickness in contrast to the limited extent of the perisomatic terminals in the *z*-axis. (g) Iba1^+^ cells (arrows) some of which surround a motoneuron cell body (MN). Scale bar indicates 25 *μ*m and 50 *μ*m for panels (f) and (g), respectively. (a–g) Shown are representative images from tissue sections after different treatments to illustrate the quality of each staining which was similar in all experimental groups and time points.

To label the quadriceps motor nucleus (experiments I and II), 125 *μ*l of 1% Fluoro-Gold (Fluorochrome, Denver, CO, USA) in saline was injected into the right quadriceps muscle without anesthesia of the rats (Figure 2(a)). For analysis of “preferential motor reinnervation” [23], 20 weeks after injury, the rats in experiment III were anesthetized as described above. The quadriceps and the saphenous branches were cut approximately 5 mm distal to the bifurcation. Fluoro-Ruby (tetramethylrhodamine dextran, MW 10,500, Molecular Probes/Life Technologies, Darmstadt, Germany) and Fluoro-Emerald (fluorescein dextran, MW 10,000, Molecular Probes) crystals were applied for 30 min to the proximal stumps of the quadriceps and the saphenous branch, respectively. Labeling was considered successful if no leakage of dye beyond the parafilm sheaths underlying the nerve ends was noticed after the 30 min application period. Six days later, the rats were anaesthetized and perfused with 4% formaldehyde in 0.1 M sodium cacodylate buffer, pH 7.3. The lumbar spinal cords were removed, postfixed overnight, and cut transversely (serial sections of 40 *μ*m thickness) on a cryostat (CM1850, Leica Microsystems, Wetzlar, Germany). The sections were collected on SuperFrost Plus glass slides (Carl Roth, Karlsruhe, Germany) and coverslipped using Fluoromount G (Southern Biotechnology Associates/Biozol, Eching, Germany). Counting was based on stereological principles and done on an Axiophot 2 fluorescence microscope [25].

### 2.5. Immunofluorescence

Tissue processing and staining were performed as previously described [26]. Under anesthesia (see above), the rats were perfused with 4% formaldehyde in 0.1 M cacodylate buffer, pH 7.3, for 15 min at room temperature (RT). The lumbar spinal cords were then postfixed in the same fixative overnight at 4°C and cryoprotected by infiltration with 15% sucrose in cacodylate buffer for 2 days at 4°C. The samples were frozen in precooled 2-methyl-butane (isopentane, −80°C) for 2 min and stored in liquid nitrogen until sectioned. Transverse sections of 25 *μ*m thickness were obtained using a cryostat (CM1850, Leica Microsystems, Wetzlar, Germany) such that 6 spaced serial sections 250 *μ*m apart were present on each slide. Immunofluorescence staining was performed after antigen retrieval (30 min at 80°C in 10 mM sodium citrate solution, pH 9.0). Nonspecific binding was blocked for 1 hour at RT with phosphate-buffered saline (PBS, pH 7.3) containing 0.2% Triton X-100 (Sigma), 0.02% sodium azide (Sigma), and 5% normal serum (Jackson ImmunoResearch Europe, Suffolk, UK) from the species in which the secondary antibody was raised (Table 1). The primary antibodies were diluted in PBS containing 0.5% lambda-carrageenan (Sigma) and 0.2% sodium azide and applied to the sections for 3 days at 4°C (Table 1). Cy3-conjugated secondary antibodies, diluted in PBS containing 0.5% lambda-carrageenan and 0.2% sodium azide, were applied for 2 hours at RT (Table 1). Cell nuclei were stained for 10 min at RT with bis-benzimide solution (Hoechst 33258 dye, 5 *μ*g ml^−1^ in PBS, Sigma). For each antigen, all sections were stained in the same primary and secondary antibody solutions stabilized by the nongelling vegetable gelatin lambda-carrageenan and kept in screw-capped staining plastic jars (capacity 35 ml, 10 slides, Carl Roth). This method enables repeated long-term usage and high reproducibility of the immunohistochemical staining [26–28]. Staining controls included omitting the first antibody or replacing it by normal serum or IgG. These controls were negative. Examples of immunohistochemical stainings are shown in Figures 2(b)–2(g).

### 2.6. Quantitative Immunohistochemical Analyses

Quantitative analyses were performed using the Stereo Investigator 8.1 software (MicroBrightField Europe, Magdeburg, Germany) and a fluorescence microscope (Axioskop 2 mot plus, Zeiss, Oberkochen, Germany) equipped with a motorized stage (Zeiss) and a CX 9000 digital camera (MicroBrightField) as described [9, 12]. Cell and synaptic terminal densities were estimated using the optical disector in every 10th spaced serial section (250 *μ*m apart) in which back-labeled femoral motoneurons were visible (Figure 2(a)). The boundaries of the quadriceps motor nucleus were outlined (Plan Neofluar 5x objective, Zeiss, Figure 2(a)), and cell or synaptic terminal densities (*N*_v_) were estimated using randomly placed disectors. For VGAT^+^ (Figures 2(b) and 2(c)), VGLUT1^+^ (Figure 2(d)), and VGLUT2^+^ terminals (Figure 2(e)), the disectors had a 100 *μ*m^2^ base and a 5 *μ*m height with an interdisector spacing of 100 *μ*m. Individually discernible immunopositive puncta were counted using a Plan Neofluar 100x oil objective (Zeiss). For Iba1^+^ cells (Figure 2(g)), the size of the disectors was 3600 *μ*m^2^ base and 10 *μ*m height and the spacing between disectors was 100 *μ*m.

Analyses of cholinergic perisomatic terminals were performed on ChAT-immunostained sections using the Stereo Investigator (Figure 2(f), [9]). All motoneuron profiles with discernible nucleus in a quadriceps motor column transect were analyzed. Each motoneuron, visualized at 100x magnification, was focused at the level of its largest cell body cross-sectional area, and its cell body perimeter and number of perisomatic terminals were determined (Figure 2(f)). Frequency of perisomatic ChAT^+^ terminals was calculated as number of perisomatic terminals per unit perimeter length. Mean values of individual animals were used to calculate group mean values.

### 2.7. Statistical Analyses

Data were analyzed using one-way analysis of variance (ANOVA) or two-way ANOVA for repeated measures followed by Holm-Sidak multiple comparison tests (SigmaPlot 12, SPSS, Chicago, IL, USA). Regression analyses were performed using SigmaPlot. The threshold value for acceptance of differences was 5%.

## 3. Results and Discussion

### 3.1. Short-Term Effects on Synaptic Terminal Numbers

**Figure 3:**
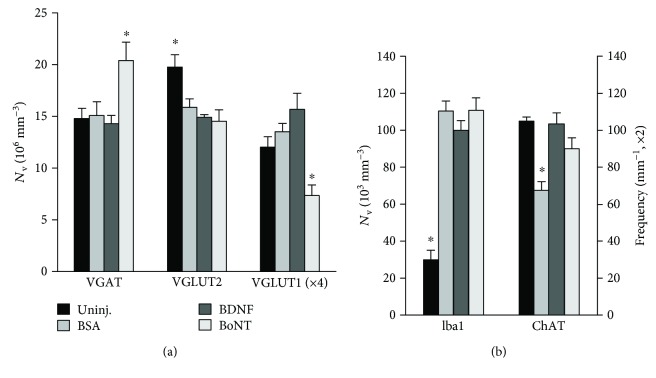
Analysis of synaptic terminals and microglia in the quadriceps motor nucleus 1 week after femoral nerve injury and drug application. Included are also values from control rats without nerve injury and drug treatment (“Uninj.”). Shown are numerical densities (number per unit volume) of VGAT^+^, VGLUT1^+^, and VGLUT2^+^ terminals and Iba1^+^ microglial cells, as well as frequency (number per unit length) of ChAT^+^ perisomatic terminals (mean values + SEM). Asterisks indicate mean values significantly different from all other groups (one-way ANOVA, *F*_3,16_ = 4.87–44.8, *p* = 0.014–<0.001) with Holm-Sidak post hoc tests (*p* = 0.042–<0.001). *N* = 5 per group.

We initially tested whether intraneural drug applications alter short-term synaptic responses to nerve injury in the spinal motor nucleus (experiment I). We estimated the effects of nerve injury and application of BSA as compared to rats without nerve lesions (“BSA” versus “Uninj.” in Figure 3) using antibodies against synaptic terminal markers (Table 1). Numbers of microglial cells were also analyzed since these cells are activated after injury and are involved in synaptic remodeling [29–31]. The observed effects included reduced density of excitatory VGLUT2^+^ terminals (−20%, Figure 3(a)), increased density of Iba1^+^ microglia (+267%, Figure 3(b)), and decrease in modulatory perisomatic ChAT^+^ terminals (−36%, Figure 3(b)). Inhibitory VGAT^+^ and excitatory VGLUT1^+^ Ia boutons were not significantly affected (+2% and +13%, resp., Figures 3(a) and 3(b)). Assuming that BSA has no measurable influence on these variables, the differences found between the two groups represent axotomy-related responses. In line with this notion is the finding of similar changes in the rat facial nucleus 1 week after axotomy [9]. Compared with BSA, BDNF had only one effect: attenuation of injury-induced ChAT^+^ terminal loss (Figure 3(b)). A similar protective effect on ChAT^+^ terminals had also BoNT (Figure 3(b)). In addition, BoNT application resulted, again as compared with BSA, in increased density of VGAT^+^ terminals (+35%) and reduced density of VGLUT1^+^ boutons (−46%), while VGLUT2^+^ terminals and Iba1^+^ cells were not significantly affected (−9% and 0%, resp., Figure 3(a)).

**Figure 4:**
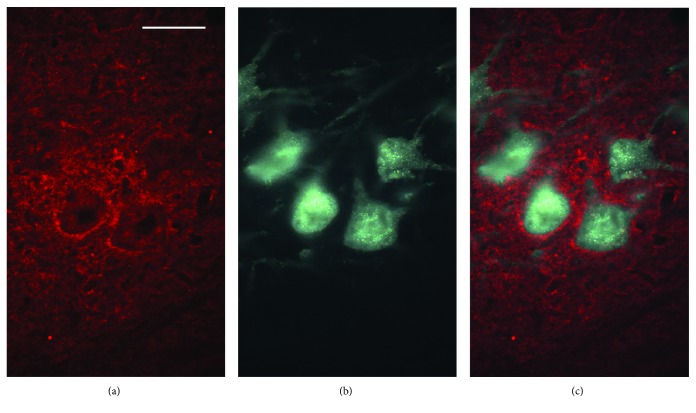
Cleaved SNAP-25 staining of a spinal cord section one week after injury and BoNT application. Immunostaining (a) is seen around the somata of back-labeled motoneurons and in the neuropil among them (b, c). Scale bar = 50 *μ*m.

To test if the BoNT effects could be related to its retrograde transport into the spinal cord, we performed immunohistochemistry for BoNT-cleaved SNAP-25 (SNAP-25_197_) which labels sites of BoNT proteolytic activity [32]. One week after nerve injury and BoNT application, immunofluorescence labeling was present around back-labeled somata and in the neuropil of the femoral motor nucleus (Figure 4). This pattern of labeling is similar to that previously observed by other groups [13, 14] and suggests that BoNT action has been transported into the spinal cord and could possibly be active in afferent terminals.

Overall, these findings show that the drug applications altered some synaptic responses to axotomy. Our working hypothesis was (see Introduction) that BDNF would have synaptotrophic effects and, indeed, injury-related loss of ChAT^+^ perisomatic boutons was prevented. At the same time, however, other major inputs, excitatory VGLUT2^+^ and inhibitory VGAT^+^ terminals, were not affected as initially hypothesized. It is possible that the intracellular concentration of active exogenous BDNF achieved in our experiment has not been optimal to produce pronounced, long-term effects. BDNF appears to have a dose-dependent influence on nerve regeneration, that is, facilitation at low doses and inhibition at higher ones [33]. Therefore, we do not assume that BDNF is inefficient in our model unless this proves true in a future dose-dependence study.

In contrast to BDNF, we expected that BoNT would enhance loss of terminals after axotomy with a more pronounced effect on excitatory (VGLUT1^+^ and VGLUT2^+^) than on inhibitory (VGAT^+^) terminals [34, 35]. This appeared true for VGLUT1^+^ terminals, but the effects on VGAT^+^ and ChAT^+^ terminals were, on the opposite, synaptotrophic (Figure 3). This heterogeneity of effects suggests also other mechanisms of action in addition to inhibition of synaptic vesicle exocytosis by cleaving SNAP-25 [35]. It is possible, for example, that the increase in inhibitory VGAT^+^ terminals results from inhibition of some of these heterogeneous in origin terminals [36] and subsequent sprouting of unaffected inhibitory axons. Partial inhibition and reactive sprouting could also affect the cholinergic input to motoneurons. Alternatively or in addition, it is possible that BoNT has neurotrophic effects achieved via colocalization and signaling through the p75 receptor [15, 37]. This notion is not necessarily in disagreement with the limited effects of BDNF described above since different receptors (p75 versus TrkB) and neurotrophins may be involved.

### 3.2. Long-Term Synaptic Effects and Recovery of Function

**Figure 5:**
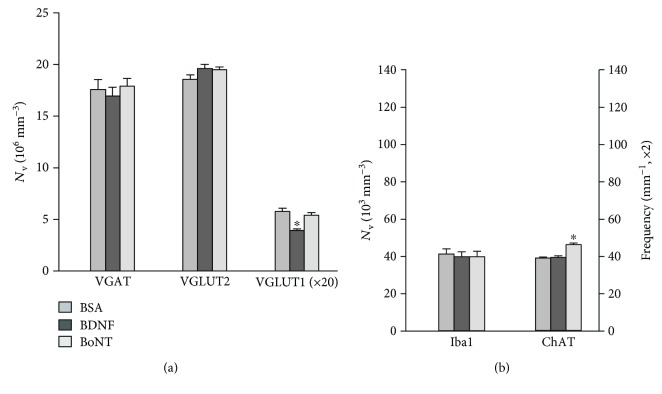
Analysis of synaptic terminals and microglia in the quadriceps motor nucleus two months after femoral nerve injury and drug application. Asterisks indicate mean values significantly different from all other groups (one-way ANOVA, *F*_2,16_ = 11.4 and 30.4, *p* < 0.002 and 0.001 for VGLUT1 and ChAT, resp.) with Holm-Sidak post hoc tests (*p* = 0.005–<0.001). *N* = 5–7 per group. Note that numbers of Iba1^+^ cells and ChAT^+^ terminals (b) and numbers of VGLUT1^+^ terminals (a) in BSA-treated animals are much lower than these at 1 week after injury (Figures 3(a) and 3(b)). This is consistent with previous findings [9, 10].

We further investigated whether drug-related synaptic alterations persist after a longer reinnervation period, two months after injury (experiment II). We found, again compared with a BSA control group, that the BDNF effect on ChAT^+^ terminals at 1-week post injury has disappeared while a previously nonexisting deficit in VGLUT1^+^ terminals was now present (Figures 5(a) and 5(b)). BoNT-related differences in VGAT^+^ and VGLUT1^+^ terminal numbers had also disappeared at two months after injury, but the ChAT^+^ terminal frequency was still higher similar to 1 week after lesion (Figures 5(a) and 5(b)). Immunohistochemistry for cleaved SNAP-25 in the spinal cord at two months after injury showed labeling similar to the one observed at 1 week (data not shown). This observation suggests that BoNT enzymatic activity is present for a long period of time after application.

**Figure 6:**
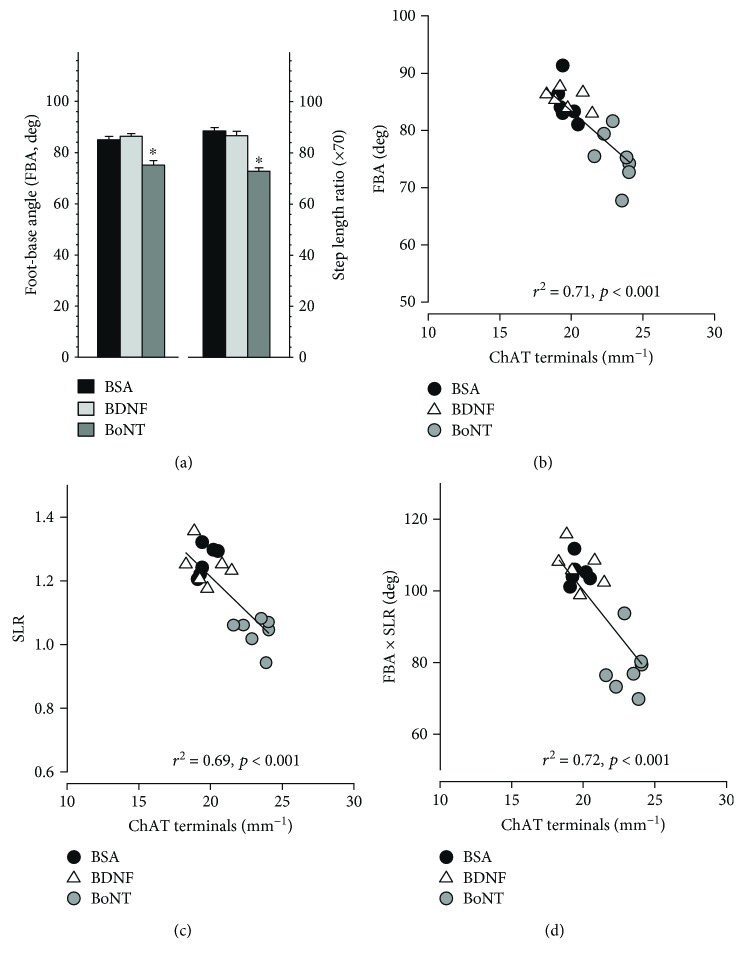
Motor recovery and correlations between functional parameters and ChAT terminal frequency two months after femoral nerve lesion and drug application. (a) Shown are mean values + SEM of foot-base angle (FBA) on the operated side and step length ratio (SLR). *N* = 6, 7, and 7 for BSA, BDNF, and BoNT, respectively. For both parameters, one-way ANOVA showed effects of treatment (*F*_2,17_ = 18.4 and 38.0 for FBA and SLR, respectively, *p* < 0.001 for both parameters). The BoNT group mean values were significantly different from the values of the BSA and BDNF groups (asterisks, *p* < 0.001, Holm-Sidak test). (b–d) Individual values of functional parameters plotted against numbers of ChAT terminals. Shown are regression lines, coefficients of determination (*r*^2^), and probability values (*p*).

**Figure 7:**
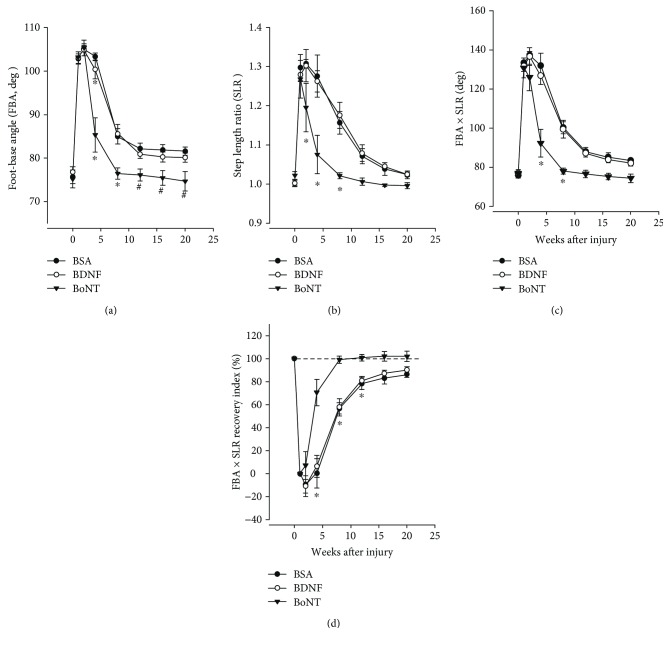
Time course and degree of motor recovery after femoral nerve lesion and drug application. Shown are mean values ± SEM of foot-base angle on the operated side (FBA, a), step length ratio (SLR, b), product FBA × SLR (c), and recovery index for the product FBA × SLR (d) prior to injury (0 week) and 1–20 weeks p.o. The dashed horizontal line in (d) is drawn at 100%, a value indicating full degree of recovery. *N* = 7, 8, and 9 for BSA, BDNF, and BoNT, respectively. For all parameters shown, two-way ANOVA for repeated measures showed effects of time (*F*_7,147_ = 52.4–209, *p* < 0.001) and treatment (*F*_2,21_ = 9.51–15.6, *p* = 0.003–<0.001). Indicated by symbols are group mean values significantly different from ^∗^ the corresponding postoperative values of the BSA and BDNF groups and ^#^ the corresponding value of the BSA group (*p* < 0.05, Holm-Sidak post hoc procedure).

Functional analysis performed in the same animal groups revealed significantly lower foot-base angle (FBA) and step length ratio (SLR) in the BoNT group as compared to BSA- and BDNF-treated rats (Figure 6(a)). This finding indicates better functional recovery as both parameters increase after injury and decrease as reinnervation and recovery proceed (see Figures 7(a) and 7(b)). Regression analysis did not indicate any significant statistical relationship between individual structural parameters (Figure 5) and functional measures (Figure 6(a)) with the exception of ChAT^+^ terminal densities (Figures 6(b)–6(d)). Higher frequencies of cholinergic perisomatic terminals appeared to be associated with lower (“better”) functional values. The coefficients of determination (*r*^2^, values shown in Figures 6(b)–6(d)) indicate that some 70% of the variability in functional parameters may be explained, in statistical terms, by variability in numbers of ChAT^+^ terminals. Previous work using facial nerve or spinal cord injury models has also shown strong statistical relationships between degree of functional recovery, on one side, and degree of preservation/recovery of ChAT^+^ terminal frequency on facial [9, 12] or spinal motoneurons [38–40], on the other side. These large cholinergic terminals form C-type synapses on motoneuronal perikarya and proximal dendrites and utilize M2 muscarinic receptors for acetylcholine in the postsynaptic membrane [41–46]. Although not that numerous, these synapses strongly influence motoneuron function by regulating action potential after hyperpolarization in a way that, under normal conditions, ensures sufficient motoneuron output to drive motor behavior [47, 48]. We can, therefore, assume that partial loss of perisomatic cholinergic terminals, associated with a reduced expression of postsynaptic receptors [49, 50], may significantly impair motor behaviors such as walking, whisking, and blinking [51].

### 3.3. Long-Term Functional Effects

Finally, we were interested whether functional effects of drug application could appear later or earlier than the analyzed postinjury time point (two months), a time period when reinnervation and recovery are well advanced but not completed. We performed experiment III in which rats were treated similarly to experiment II but monitored functionally between the first and the 20th week after injury. Time course and degree of recovery were very similar between BSA- and BDNF-treated animals (Figures 7(a)–7(d)) and in agreement with previous observations after transection and suture of the femoral nerve in adult rats [23]. In contrast, recovery after BoNT application was accelerated between the 2nd and 12th week (Figures 7(a)–7(d)) and advantages of this treatment were even present at the final time point studied, 20 weeks (Figure 7(a)).

**Figure 8:**
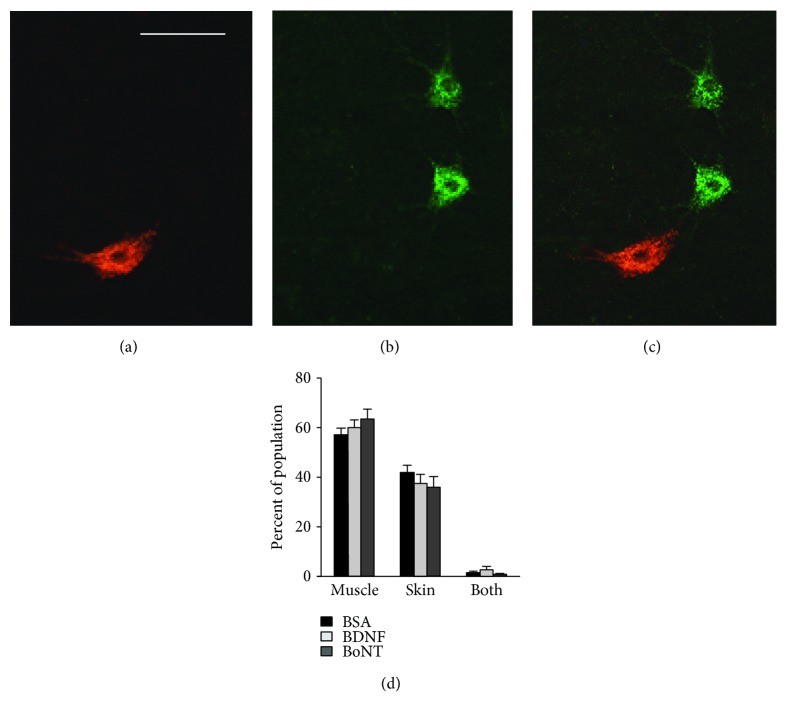
Retrograde labeling of motoneurons 20 weeks after lesion. (a–c) Representative images of motoneurons back-labeled through the muscle (quadriceps) and the skin (saphenous) branch of the femoral nerve (“Muscle” and “Skin”) using Fluoro-Ruby and Fluoro-Emerald (red and green fluorescence), (a) and (b), respectively, overlay in (c). Scale bar = 100 *μ*m. (d) Quantitative analysis of retrogradely labeled cells including double-labeled motoneurons (“Both”). Shown are mean values + SEM. One-way ANOVA showed no effect of treatment on any of the motoneuron categories (*F*_2,18_ = 0.95–1.14, *p* = 0.342–0.533). *N* = 7 animals per group.

After the 20-week observation period, the animals in experiment III were subjected to retrograde labeling to assess precision of reinnervation (Figures 8(a)–8(c)), a factor that can influence the functional outcome after femoral nerve injury and regeneration in rats [23]. The numbers of motoneurons projecting into the appropriate quadriceps nerve only, into the inappropriate saphenous nerve, or into both nerves (“Muscle,” “Skin,” and “Both” in Figure 8(d), resp.) were similar in the three groups of rats. This finding suggests that the functional improvements seen in the BoNT group are not related to an enhanced preferential reinnervation of the muscle. This notion is supported by the lack of significant covariations between numbers of back-labeled motoneurons and functional parameters.

### 3.4. Possible Mechanisms of Drug Effects

We applied BoNT only once using the time frame between axonal membrane damage and sealing to load the proximal axon and cell body with toxin similar to retrograde tracers (Figures 8(a)–8(c)). Our expectation was that this uptake will be sufficient to “prime” the initial responses of motoneurons to injury, in particular their deafferentation, and, thus, eventually achieve long-term effects on regeneration without need of repeated drug delivery to the injury site. As estimated by gait analysis, our experiment was successful as functional regeneration was enhanced already at two weeks after injury and recovery remained accelerated for months thereafter. Enhancement of axonal regrowth in the crushed sciatic nerve of mice by a single low-dose intraneural application of BoNT has been just reported, but the underlying mechanisms for these effects have remained unclear [37]. Here, we propose that the improvement of regeneration in our model is a consequence of attenuated loss of cholinergic modulatory input to femoral motoneurons (Table 2). In addition, it is possible that BoNT has an additional neuroprotective effect. At one week after injury, we found, compared with control rats, an increase in VGAT^+^ inhibitory afferents in the quadriceps motor nucleus, reduced numbers of excitatory VGLUT1^+^ Ia afferents, and no change in excitatory VGLUT2^+^ terminals (Figure 3, Table 2). We can speculate that this constellation attenuates the increased excitability of the axotomized motoneurons and, thus, allows better recovery of the motoneuron and its better regeneration [18, 52]. It is also thinkable that BoNT-related modulations of reflexes and/or pain-related transmission may have also positive functional consequences [53–56]. A major unresolved issue in this study is why BoNT had synaptotrophic effects on some types of synapses. The unexpected observation, which is unrelated to the main goal and achievement of this work, has to be explained by future experiments.

Similar to BoNT, BDNF is retrogradely transported from the periphery to the cell body of motoneurons and then transcytosed to afferent presynaptic terminals [57]. Exogenous BDNF has already shown synaptotrophic properties in injury models [18, 19, 58], and exogenous BDNF can improve axonal regeneration [59, 60]. We indeed found a BDNF effect at one week after injury—prevention of injury-induced ChAT^+^ terminal loss (Figure 3, Table 2), but no functional effects were seen (Figures 6(a) and 7). This may be related to lack of a prolonged protective effect on ChAT^+^ terminals as observed two months after BoNT application (Figure 5, Table 2).

## 4. Conclusions

The results of this study provide further support to the notion that insufficient recovery of synaptic inputs to motoneurons, in particular, perisomatic cholinergic terminals, may be an essential factor limiting recovery after peripheral nerve injury and regeneration. In addition, it appears encouraging that single intraoperative application of drugs to the severed nerve can be a useful way to modify neuronal responses to axotomy and, thus, modulate regeneration and eventually improve functional outcome of nerve injury. The list of candidates for such applications may be long, ranging from other neurotrophins or combinations of neurotrophins (e.g., BDNF and neurotrophin-3 [18], NGF [61]) or growth factors (e.g., vascular endothelial growth factor (VEGF) [62]) to small bioactive molecules [63].

## Figures and Tables

**Table 1:** Antibodies used for immunohistochemistry.

Antigen	Species and type, dilution	Supplier, code	Structures labeled by primary antibodies	References
Choline acetyltransferase	Goat polyclonal, 1 : 500	Chemicon/Millipore, Schwalbach, Germany, AB144P	Cholinergic cells, axons and axon terminals, large perisomatic terminals on motoneurons	Hellström et al. [44], Nagy et al. [45], Wilson et al. [46]
Iba1 (ionized calcium binding adaptor molecule 1)	Rabbit polyclonal, 1 : 1500	Wako Chemicals, Neuss, Germany, 019-19741	Microglial cells	Imai et al. [64], Ito et al. [65]
VGAT (vesicular GABA transporter)	Mouse monoclonal, 1 : 500	Synaptic Systems, Gottingen, Germany, 131 011	Inhibitory (GABAergic and glycinergic) axon terminals	Chaudhry et al. [66], McIntire et al. [67], Wojcik et al. [68]
VGLUT1 (vesicular glutamate transporter 1)	Rabbit polyclonal, 1 : 1000	Synaptic Systems, 135 303	Excitatory (glutamatergic) axon terminals of primary (Ia) afferents	Alvarez et al. [69], Oliveira et al. [70], Rotterman et al. [10]
VGLUT2 (vesicular glutamate transporter 2)	Rabbit polyclonal, 1 : 1000	Synaptic Systems, 135 403	Excitatory (glutamatergic) axon terminals of spinal cord interneurons	Alvarez et al. [69], Oliveira et al. [70]
SNAP-25 BoTox-A cleaved	Mouse monoclonal (4F3-2C1), 1:200	MyBioSource, San Diego, CA, USA, MBS350064	Synaptic terminals containing SNAP-25 (synaptosomal-associated protein 25) cleaved by botulinum toxin A	Manufacturer's data sheet, Rheaume et al. [32]
Goat IgG	Cy3-conjugated donkey polyclonal, 1 : 200	Jackson ImmunoResearch Europe, Suffolk, UK, 705-165-003		
Mouse IgG	Cy3-conjugated goat polyclonal, 1 : 200	Jackson ImmunoResearch, 115-165-003		
Rabbit IgG	Cy3-conjugated goat polyclonal, 1 : 200	Jackson ImmunoResearch, 111-165-003		

**Table 2:** Summary of effects of drug application on VGLUT1^+^, VGLUT2^+^, ChAT^+^, and VGAT^+^ synaptic terminals and Iba1^+^ cells one week and two months after injury. Arrows indicate increase (↑), decrease (↓), or no difference (=) compared to BSA treatment.

	BoNT versus BSA		BDNF versus BSA	
	1 week	2 months	1 week	2 months
VGLUT1	↓	=	=	↓
VGLUT2	=	=	=	=
ChAT	↑	↑	↑	=
VGAT	↑	=	=	=
Iba1	=	=	=	=

## Data Availability

The data used to support the findings of this study are available from the corresponding author upon request.
